# An overview of cybersecurity in Zimbabwe’s financial services sector

**DOI:** 10.12688/f1000research.132823.1

**Published:** 2023-09-29

**Authors:** Vusumuzi Maphosa

**Affiliations:** 1 Department of Information and Communication Technology Services, National University Science and Technology, Bulawayo, Bulawayo, 263, Zimbabwe

**Keywords:** cybersecurity, cybercrime, threats, barriers, frameworks

## Abstract

**Background:** As nations, businesses, and individuals rely on the Internet for everyday use, so are cybercriminals manipulating systems to access information illegally and disrupting services for financial gain. The global cost of cybercrime eclipsed one trillion US Dollars in 2020, with Africa losing US $3.5 billion.

**Methods:** A quantitative research methodology was adopted to investigate factors affecting cybercrime in Zimbabwean financial institutions. The study focused on the technical aspects of cybersecurity. Data were collected from July 2022 to October 2022, targeting technology experts in the financial services sector. Participants were recruited from 13 institutions to rank cybersecurity constructs, frameworks, and challenges associated with cybersecurity. Data was collected using a questionnaire distributed to participants. Descriptive statistics were used to extract meanings from the responses that measure mean and standard deviation.

**Results:** Network and data security were the most highly ranked cybersecurity constructs, while physical security was the least. The top three barriers are increasing sophistication of threats, limited skills and emerging technologies, while lack of executive support was the least. The top frameworks used are the Information Technology Infrastructure Library (ITIL) and Control Objectives for Information and Related Technologies (COBIT), while a fifth is yet to adopt cybercrime frameworks.

**Conclusions:** The study proposes that financial institutions establish a cybersecurity culture to fight cybercrime, addressing cybersecurity barriers and following best practices. Financial institutions should invest in cybersecurity technologies, train security specialists, and employ a Chief Information Security Officer (CISO). The study’s small sample may affect the generalisability of the results. Financial institutions should implement strategies to raise awareness and collaborate with institutions to train cybersecurity security specialists to close the skills gap.

## Introduction

The internet supports today’s knowledge economy and affects every way of life. The internet is growing in volume and complexity, and without due care, it exposes private data and information to criminals. Businesses use information and communication technologies to drive production and automation, while consumers use technology for entertainment and services ( Maphosa, 2022). The International Telecommunication Union (ITU) reports that fixed broadband access has increased globally. Decreasing data costs have fueled teleworking, electronic commerce (e-commerce), distance learning, remote entertainment, and telemedicine ( Katz and Jung, 2021). The over-dependency on information technology (IT) systems and the rise of e-commerce make societies more vulnerable to cyber-attacks than ever. Nations, institutions, and individuals utilise cyberspace, a digital or virtual environment where they connect and access digital resources. Devices, networks, and systems that harness information and knowledge for economic development should be secured. Cyberattacks disrupt services and critical infrastructure such as electric grids, water supply and transport systems, banking, and social network systems. Cybersecurity breaches impact national security, economies, and individual livelihoods as attackers leverage the vulnerabilities on multiple devices connected to the internet.

The cost of cybercrime and cybersecurity in 2020 exceeded 1% of the world’s gross domestic product eclipsing one trillion US dollars ( Sviatun *et al.,* 2021). New forms of businesses purely mediated by the internet, such as crypto-currencies, virtual reality, and cloud computing, have emerged. The adoption of new technologies such as artificial intelligence, blockchain, and the Internet of Things (IoT), among others, offers the hacker community new methods and skills to breach and undermine the security of organisations that suffer irrecoverable losses ( Radzikowski, 2015). When systems have been compromised, attackers may lock the data illegally and demand ransoms to restore compromised data. The internet provides significant advantages as customers can flip through virtual online systems to acquire goods and services. However, criminal elements are lurking, intercepting, and tracking these transactions for fraud. Industry experts report that IoT devices will surpass 75 billion units by 2025 ( Hejase *et al.,* 2021), offering more opportunities for cybercriminals to breach insecure systems and homes and access sensitive data.

Cyberspace comprises of three layers: infrastructure, software, and data. The infrastructure layer includes physical devices and network equipment, the software layer includes computer systems and applications, and the data layer includes the data held in storage devices. To reduce the impact of cyberattacks, institutions harden cyber resources through software upgrades and patches and train employees, constantly identify vulnerabilities, and mitigate effects through backups. Institutions deploy solutions to protect their cyber resources, from physical security to application and data security. Physical cybersecurity refers to using biometric controls, physical locks, alarm systems, human security guards, and video surveillance cameras to safeguard the tangible cyber assets of an organisation ( Goldstein, 2016). Cybersecurity controls and measures are applied to an organisation’s cyber applications to reduce the risk of breach. Data cybersecurity protects the confidentiality, integrity, and availability of cyber data to meet the data user’s requirements. Network security safeguards systems against unauthorised access. Network cybersecurity refers to measures taken to protect data during transmission over interconnected networks (Awodele, Onuiri, and Okolie, 2012). Network cybersecurity entails enforcing policies and modifying the network architecture to include security controls such as firewall rules, intrusion detection, monitoring, and patch management.

Identity theft is the illegal use of another person’s private information for fraud. It involves impersonating an individual’s identity to steal personal information, including banking details, credit cards, and social security information ( Arachchilage and Love, 2014). Phishing is a social engineering technique where an attacker seeks to access a legitimate user’s credentials illegally or personally sensitive information by impersonating electronic communications from a trusted source ( Jakobsson and Myers, 2006; Jang-Jaccard and Nepal, 2014). During phishing, an unsuspecting victim is redirected to a malicious website after receiving an email with an embedded link using social engineering techniques ( Gupta *et al.,* 2015). Malware refers to software programs illegally installed on a victim’s computer to steal identifying information and cause malicious damage to cyberinfrastructure.

As banks go digital, customers use electronic devices to conduct banking services such as creating accounts, conducting financial transactions and paying bills anytime and anywhere, increasing exposure to cybercrime. Most of Africa’s economy is informal; therefore, cyberattacks target financial institutions and mobile network operators who drive the mobile money ecosystem ( Mukiibi, 2019). Africa loses over four billion USD annually to cybercrime; other critical losses include data, intellectual property, reputation, and brand name ( Weforum, 2022). The outbreak of the COVID-19 pandemic forced organisations to shift from the physical to the virtual environments to deliver services ( Maphosa, 2021), putting a strain on cybersecurity. Cybercrimes continue to increase despite the availability of technical cybersecurity infrastructures such as firewalls, encryption, and antiviruses.

Developing countries should strengthen cybersecurity measures as attacks on critical infrastructure are rising. According to the national cybersecurity index (CGI), a global tracker of countries’ progress in cybersecurity, Zimbabwe is ranked 129 ^th^ due to a lack of policies that support the cybersecurity (NCSI, 2021). Cybersecurity breaches are rising in Zimbabwe due to a lack of a national cybersecurity implementation plan and strategy in Zimbabwe (NCSI, 2021). In a 2018 survey, 64% of industry leaders acknowledged that organisations had failed to manage cybersecurity risks; therefore, improvements were proposed ( Deloitte, 2018). This calls for researchers to propose and evaluate technical cybersecurity solutions for combating cybercrime. The study aims to assess the state of cybersecurity in a developing country to raise awareness and compliance and fight cybercrime. The study also adds to the dearth of literature from developing countries on cybercrime.

### Literature review

Cyberspace is the fastest evolving technology in human history, where new emerging platforms such as IoT, social media, big data, and cloud computing provide new threats and opportunities. Despite the recent adoption of digital platforms in Africa, organisations still need to prioritise cybersecurity; unfortunately, only a few have developed comprehensive policies to improve security. Criminals have expanded their attacks as many systems are vulnerable due to lax cybersecurity practices in most African countries. Mukiibi (2019) reported that less than ten African countries have cybersecurity legislation. By 2022, 29 of the 54 African countries had cybersecurity legislation ( Weforum, 2022). In 2018, only 13 of the 54 African countries had Computer Emergency Response Teams (CERT), and 14 had personal data protection laws ( AUC, 2018). Mukiibi (2019) reports that only 18 countries have Computer Security Incident Response Teams (CSIRTs). As a result, many organisations are vulnerable, and assessment results revealed that only 52% of African companies could handle large-scale cyber-attacks ( Weforum, 2022). In the 12 months ending February 2021, South Africa had 230 million attacks, followed by Kenya and Morocco, which recorded 72 and 71 million attacks, respectively ( KPMG, 2022). Interpol reports that 90% of African businesses have no cybersecurity protocols to protect their businesses, leaving them vulnerable to threat actors (Scidenet, 2016). Zimbabwe and Libya had 90% of counterfeit and pirated software, the highest percentage, accelerating the spread of malware and system breaches (Scidenet, 2016; Kshetri, 2019).

### Cybercrime

Cybercrimes are known as crimes of the Internet; specifically, they refer to criminal activities perpetrated through computer-related devices in cyberspace ( Kharb, 2017). As more workers took their computers to work from home during the COVID-19 pandemic, industry experts report that cyberattacks quadrupled (Menn, 2020). The World Economic Forum reports that cyberattacks increased to 125% globally in 2021, and indications show an upward increase in 2022 ( Weforum, 2022). Ever since the outbreak of the COVID-19 pandemic, cybercrimes have increased by 300%, costing the world over six trillion USD ( Hejase *et al.,* 2021). Sviatun *et al.,* (2021) reported that 87.6% of cybercrime attacks targeted the financial sector, with the retail industry coming second with 82.7%, while the communication and technology sector had 81.9%. African businesses face cyber threats such as online scams, ransomware, botnets and email compromise ( KPMG, 2022). The most common cybercrimes in Zimbabwe are identity theft, hacking, email phishing, and malware victimisation ( RBZ, 2015).

A study carried out by Kahn and Roberds (2008) showed that identity theft was driven by the need to steal money on one side and the need to avoid being monitored. Alkhalil *et al.,* (2021) postulated that phishers attack a technical system by tricking employees into clicking on malicious links or downloading harmful files to steal their private information required to commit fraud. Molinaro and Bolton (2018) highlighted the importance of the double lens model in preventing phishing attacks. Hacking has been attributed to low self-control ( Kranenbarg, Holt, and Gleder, 2017). Odunze (2018) employed the differential association theory and the routine activity theory to explain hacking and found that women were more vulnerable to hacking than their male counterparts due to the prevalence of romance scams.

### Cybersecurity

Cybersecurity combines procedures and processes to protect infrastructure, systems, and data from cyberattacks. Cybersecurity ensures data integrity and confidentiality by guarding against unauthorised access to sensitive information ( Mukiibi, 2019). Cybercriminals exploit flaws and other vulnerabilities in emerging technologies to counter security offered by firewalls, antivirus scanners, and data encryption tools ( Jang-Jaccard and Nepal, 2014). On average, organisations are paying US $3.6 million per attack, with ransomware attacks increasing by 151% as organisations witnessed a 31% increase in attacks ( Bissell *et al.,* 2021). A major cyber-attack on a power grid left over 1.4 million people without electricity in Ukraine ( Knake, 2017). Financial institution systems have become a significant target for hacking, phishing, malware, and identity theft (Scidenet, 2016). Industry trends show exponential cyber-attack growth; Price Waterhouse Cooper (PWC) reported that 93% of financial institutions suffered security breaches in 2016 ( Airehrour, Vasudevan, and Madanian, 2018). The financial services sector can become bankrupt after a security breach, with millions of dollars demanded to pay lawsuits and settle ransomware ( Reddy and Reddy, 2014). After a phishing attack in 2017, the Bank of India lost US $170 million ( Acharya and Joshi, 2020). Another bank in Brazil lost US $243 million to cyber criminals ( Tabassum, 2020). Industry experts reported that Africa lost over US $3.5 billion in 2017, with Nigeria accounting for 18.5% (US $649m), Kenya losing 6% (US $210m), and South Africa losing 4.5% (US $157m) through cyberattacks ( Kshetri, 2019). Klynveld Peat Marwick Goerdeler (KPMG) reported that Kenya’s interconnected supply chain networks had suffered ransomware attacks ( KPMG, 2022). In contrast, its banking sector has suffered from distributed denial-of-service (DDoS) attacks. Cyber threats have disrupted South Africa’s maritime infrastructure, and its cities’ social services payment systems have suffered ransomware and data breach ( KPMG, 2022). In 2018, over 4,000 cases of cybercrime were handled by Zimbabwean police, and the country lost US $40 million to cybercrime in 2018 ( Bulawayo24, 2021).

Cybercriminals target and exploit technical vulnerabilities and pry on users with limited cyber training or ethics to breach systems. Physical security is achieved using human guards, video surveillance cameras, physical locks, and biometrics to protect cyberspace. Skopak and Sakanovic (2016) confirmed that physical security is necessary to protect information resources comprehensively. Kazemi (2018) asserted that physical security was among the factors helping to preserve confidentiality. This view was supported by Elnaim (2016), who found out that physical security helped to protect information against attacks. DiMase *et al.,* (2015) highlighted the importance of physical security in denying access to hardware resources. Georgiadou *et al.,* (2021) reported that it was easy to control machines as they were more predictable than humans. There is growing interest and broader emphasis on human factors in the fight against cybercrime.

As financial institutions move some of their services online, potential breaches and security attacks increase exponentially. Reaves *et al.,* (2015) analysed branchless banking applications and reported increased cybersecurity threats. ENISA (2016) showed that application security influences cybersecurity. Elkhodr *et al.,* (2012) proposed improving mobile banking’s application security in Australia and found that mobile application security significantly impacted cybersecurity. Ahluwalia (2016) postulated that biometrics were pivotal in mitigating cybersecurity breaches. Experimental results from a study conducted by Zhang and Wang (2010) showed that network security performance contributed to cybersecurity.

Globally, internet traffic increased by over 30%, with significant changes in geographic distributions of the connections from enterprise locations to residential access ( Katz, 2020). As workers move to work from home due to COVID-19 and flexible working in line with 21 ^st^-century jobs, vulnerabilities intensify, and measures are required to protect data during transmission over interconnected networks. Gyabi and Shrivas (2016) used encryption to secure data in the rural bank of Ghana. A simulation analysis by Hossain *et al.,* (2017) revealed that data security in the cloud could be achieved through encryption and a location-based salt algorithm. Durumeric *et al.,* (2017) sought to avoid HTTPS interception through heuristics deployed on different networks. A study carried out by Subramanian and John (2017) revealed that a data security algorithm reduced malicious insider attacks. Kaiwartya *et al.,* (2017) investigated biometric Internet security and found it suitable for Internet authentication. Tseng *et al.,* (2015) realised the importance of internet security and proposed an anti-phishing-based video game to enhance the learners’ internet security.

## Methods

### Ethical statement

This study received ethical approval from the Lupane State University Institutional Ethics Committee (LSU00022). The online questionnaire explained the research objectives, participants' expectations, voluntariness and respondents’ anonymity. Participants gave their written consent before participating in the online survey.

### Study design

The study applied a descriptive quantitative survey design. The comprehensive literature review identified critical technical factors influencing cybersecurity, such as physical security, data security, application security, network security, and internet security. These factors shaped the thrust of the study. The study’s questionnaire was adapted from the International Organization for Standardization (ISO)/International Electrotechnical Commission (ISOC/IEC) ( ISOC/IEC, 2012). ISOC/IEC is a task force responsible for crafting and reviewing industry-wide cybersecurity standards after every five years. Since the targeted participants are professionals with post-secondary education, the questionnaire was administered in English, the country’s official language.

A pilot study was conducted to verify the questionnaire’s appropriateness and completeness and gauge the meaning of the questions ( Maphosa, 2023c). The instrument was piloted in June 2022 to six network and security personnel at the University. Participants made comments and suggestions on the online questionnaire, which the researcher captured. This ensured that the questions were not ambiguous, difficult to answer or prone to many interpretations, which could lead to biased responses. Before the survey instrument was disseminated, some questions were edited to ensure clarity and answerability, while some were re-arranged to improve the flow of responses. Other changes involved altering some binary responses ‘yes’ or ‘no’ to the Likert scale type and providing options to other questions.

The final questionnaire contained two key sections with 38 items based on the literature reviewed. Cybersecurity literature and security governance standards were contextualised to the Zimbabwean context to develop the questionnaire. Data were collected from early July 2022 to early October 2022. Section A contains the respondent’s age, financial institution’s name, and gender profile as shown in Appendix A. Section B consisted of the main questions on a five-point Likert scale ranging from 1 = Strongly Disagree to 5 = Strongly Agree ( Maphosa, 2023b).

### Participant selection and data collection

The study targeted IT experts from the country’s financial institutions comprising commercial banks, merchant banks, discount houses, building societies, and finance houses. The survey questionnaire for the study was self-administered to IT experts in the financial services sector to obtain an overview of cybercrime. Electronic mail was sent to personnel in the networks and infrastructure departments of the randomly selected financial institutions. Professional networks such as LinkedIn and Twitter and distribution lists such as the Computer Society of Zimbabwe and the Internet Society of Zimbabwe were used. Convenience sampling was used to recruit participants. The sample includes participants working in the networks and infrastructure department within their financial institutions. Data were collected electronically and stored in Google Drive, which was password encrypted.

## Results

The 76 responses received were from ICT managers (16), network security specialists (12), database and systems administrators (10), developers (28), and risk and compliance officers (10), giving an 84.4% response rate. Most (72%) respondents were male, while about a third were female. More than half of the respondents had an undergraduate degree, as shown in Table 1. The average age of the respondents was 29 years, while the IT experience in the financial services sector was 9.5. The respondents’ names and those of their financial institutions were kept anonymous ( Maphosa, 2023a).

**Table 1.** Demographics of the 76 participants.

Demographic variables	Demographic variables values
Gender	Male (72%); Female (28%)
Age	Mean 29; range 19-55 years
Qualification	Diploma (14%), undergraduate (51%), postgraduate (35 %)
IT experience in a financial institution	Mean = 9.5 years, range 1-40 years
Occupation	Developers, Database administrators, Systems administrators, Network security specialists, Managers, and Risk compliance officers

The study evaluated cybersecurity security constructs for initial threat areas from physical security to data security. The study used descriptive statistics and percentages to measure the constructs’ means and standard deviations (SD). The mean and the SD of the cybersecurity constructs are shown in Table 2. The mean values ranged between 3.699 and 4.854, while SD values ranged between 0.655 and 0.779. Participants ranked network security highly, with a mean of 4.854 and a standard deviation of 0.739. The following ranked construct was data security, with a mean of 4.739 and an SD of 0.655. The penultimate construct was identity theft, with a mean of 3.802 and an SD of 0.715. The last ranked construct was physical security, with a mean of 3.699 and an SD of 0.770. Physical security is easily fortified through security guards, CCTV, biometrics, electronic locks or other related devices.

**Table 2.** Security constructs and ranking.

Constructs	Items	Mean	SD
Physical Security	5	3.699	0.770
Application Security	5	4.731	0.779
Data Security	4	4.739	0.655
Network Security	4	4.854	0.739
Internet Security	5	3.964	0.703
Identity Theft	4	3.802	0.715

Respondents ranked the framework used by their financial institution; almost half (44.74%) used the Information Technology Infrastructure Library (ITIL), followed by Control Objectives for Information and Related Technology framework (COBIT), with 36.84% and about 10.53% of the institutions adopted other frameworks as shown in Table 3.

**Table 3.** Frameworks: cybersecurity.

Cybersecurity Framework	Frequency	Percentage
ITIL	34	44.74%
COBIT	28	36.84%
NIST	19	25%
ISO/IEC27000	17	22.37%
No framework used	15	19.74%
Others	8	10.53%

**Figure 1.**
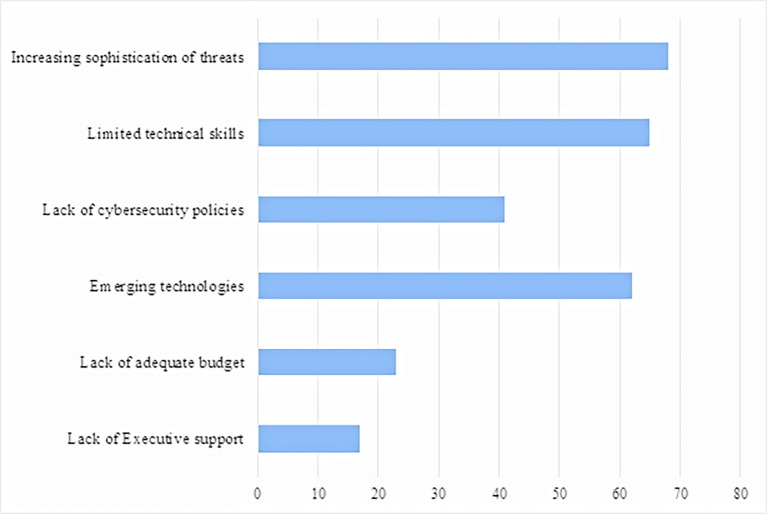
Barriers to cybercrime management.

Figure 1 shows the top cybersecurity barriers, which include increasing sophistication of threats (89.5%), limited technical skills (85.5%) and emerging technologies (81.6%). The least ranked barriers are lack of executive support (22.4%), lack of adequate budget (30.3%) and lack of cybersecurity policies (53.9%).

## Discussion

This study confirmed the findings by Norris *et al.,* (2019), who established that lack of skills, inadequate policies, limited funding and management support impacted cybersecurity. The results show that about a fifth (19.74%) of the financial institutions have yet to adopt cybercrime frameworks, worrying as public funds and investments are at risk. Institutions must balance the drive to increase revenues and reduce operating costs while ensuring compliance and investing in cybersecurity frameworks. The ranking of network security aligned with findings by Praveena and Smys (2017), who identified network security as a substantial concern in protecting financial information. Acharya and Joshi (2020) also contended that networks should be audited at fixed intervals to test for security breaches.

Bendovschi (2015) also ranked data security highly, noting that when organisations lose their data, they lose their market share and customer relationship. Figure 1 shows the barriers to cybercrime in line with Norris *et al.,* (2019), who ranked cybersecurity management barriers as limited technical skills caused by the inability of institutions to pay competitive salaries. Verizon reports that 37% of security breaches resulted from identity theft, while social engineering or phishing accounted for 22% ( Verizon, 2020). KPMG (2022) recommended that organisations perform penetration tests regularly and demonstrate response and readiness to evaluate the institution’s network security. The minimal technical skills mean institutions have limited capacity to secure networks and information systems, configure servers, recover data, and continuously scan for vulnerabilities and remediation.

Cavusoglu *et al.,* 2004 lamented that most African countries needed meaningful budgets to support cyber security. The establishment of cybersecurity policies demonstrates management’s intent to create a culture and provide a guide to employees. Although management support was lowly ranked, none of the financial institutions had established a Chief Information Security Officer (CISO) to handle cybersecurity issues at a strategic level. Management support also influences budgetary allocations and prioritises cybersecurity issues within the institution. Richards (2014) noted that organisations had established the CISO to strengthen the institution’s cybersecurity portfolio, managing enterprise cybersecurity risks and mitigation measures to maintain the institutional brand.

Cybercriminals rely on sophisticated technologies that are difficult to detect and threaten even the savviest targets ( Microsoft, 2020). The findings align with observations by the World Economic Forum, which reported that only 53.7% of African countries had cybersecurity policies ( Weforum, 2022). The lack of policies hinders sharing cybersecurity information between institutions, resulting in fragmented knowledge across the domain. Caulkins *et al.,* (2018) also identified a lack of cybersecurity personnel globally, affecting the availability of skilled and experienced staff who can handle cybersecurity tasks and challenges. Financial institutions should train and retain cybersecurity specialists to fight cybercrime.

Microsoft (2020) identified emerging threats, such as using AI-enabled capabilities to commit cybercrime and the increased adoption of IoT and teleworking. Such tools are available on the black market and online. Financial institutions can take practical steps to raise awareness and training and ensure that cybersecurity frameworks are adopted. Lack of cybersecurity awareness by employees can have devastating consequences on the organisation as they can quickly become a security loophole if they are not concentrating, are distracted, or are stressed. This aligns well with KPMG's (2022) recommendations, which suggested that institutions conduct cyber awareness and training, establish firewalls and maintain backup while ensuring their security systems have the latest patches. The top three barriers for this study are increasing sophistication of threats, limited skills and emerging technologies. Insufficient cybersecurity personnel, limited budgets, and executive support followed these. Financial institutions should adopt cybersecurity policies and engage in extensive end-user training programmes to fight cybercriminals.

The study has the following limitations. The small sample size impacts the generalisability of the findings; more responses would have improved the value of the study’s findings. Using a quantitative data collection approach may have restricted the probing of participants to elicit more information and further explain specific responses. The use of self-reported data raises fears that participants could have portrayed a positive outlook on the image of their institution since data breaches are sensitive in the financial services sector.

## Conclusion

Literature shows that cybercriminals constantly attack financial institutions, yet results show that their cybersecurity practices are poor. As technology evolves, the means and opportunities to commit cybercrime also increase, and therefore, many organisations will suffer security breaches leading to irrecoverable losses. The study provides an overview of Zimbabwe’s cybersecurity landscape and threats while providing a roadmap to manage cybercrime in other developing countries with a similar socioeconomic environment. Research has been conducted to identify the motivations, techniques, and countermeasures to cybercrime; however, there is no single solution due to the heterogeneous nature of the attack vector. Financial institutions should embrace a strong awareness culture, invest in cybersecurity technologies, train security specialists, and employ CISOs and executives knowledgeable in cybersecurity.

The study established technical factors such as physical security, application security, data security, network security, and internet security. Network security and data security were the highly ranked cybersecurity constructs, while physical security was the least ranked. There are several barriers that financial institutions face in managing cybercrime. The top three barriers are increasing sophistication of threats, limited skills and emerging technologies. The top frameworks used by financial institutions are the ITIL and COBIT, while about a fifth are yet to adopt cybercrime frameworks. The study’s small sample may affect the generalisability of the results. The study focused on technical aspects of cybersecurity, and future studies could focus on social engineering aspects that compromise the security of systems. This study raises awareness of the ever-present cybersecurity threat in the financial services sector. The study provides a baseline on the state of cybercrime in developing countries. More research will be required to validate these findings by developing models and using advanced statistical analysis on independent and dependent variables to test for causality and correlation.

The Government should proactively provide an environment that supports cybersecurity research and reporting of cases so that institutions can learn from others and continuously improve their detection and protection systems. The study recommends developing a national cybersecurity framework for an improved cybersecurity strategy for protecting Zimbabwean financial institutions. This framework must include establishing a cybersecurity culture, addressing cybersecurity barriers and following best practices such as adopting frameworks and establishing the office of the CISO. This will improve the protection of critical assets, minimise service disruption and loss of resources and strengthen financial institutions’ public confidence and reputation. The practical implication of this study is improving cybersecurity risks, given the rising adoption of emerging technologies and frameworks that support participation in the global economy.

Future studies could investigate the effects of social media-based cybercrimes as emerging threats are predicted to increase significantly over the following years. The government should also implement and strengthen policies, laws and legislations that curb cybercrime to mitigate economic losses. Financial institutions must create strategies to raise awareness of cybercrime and collaborate with higher education institutions to introduce programmes addressing cybersecurity challenges to close the skills gap. Financial institutions can use social media platforms for cybersecurity literacy and awareness.

## Data Availability

Zenondo: Cybersecurity. https://doi.org/10.5281/zenodo.7824605. ( Maphosa, 2023a). The project contains the following underlying data:
• Cyber security survey.xlsx. (Anonymised responses from IT experts on cybersecurity in the financial services sector). Data are available under the terms of the Creative Commons Attribution 4.0 International license (CC-BY 4.0). Zenondo: An Overview of Cybersecurity in Zimbabwe's Financial Services Sector. https://doi.org/10.5281/zenodo.7824658. ( Maphosa, 2023b). This project contains the following extended data:
• Cybersecurity questionnaire.pdf. (Final version of the cybersecurity survey questionnaire in the financial services sector). Zenondo: An Overview of Cybersecurity in Zimbabwe's Financial Services Sector. https://doi.org/10.5281/zenodo.7825562. ( Maphosa, 2023c).
• Questionnaire pilot.pdf (Pilot version of the Cybersecurity survey questionnaire in the financial services sector). Data are available under the terms of the Creative Commons Attribution 4.0 International license (CC-BY 4.0).
