# Supplementary material for: An overview of cybersecurity in Zimbabwe’s financial services sector
Source: F1000Res. 2024 Mar 14;12:1251. Originally published 2023 Sep 29. [Version 2] doi: 10.12688/f1000research.132823.2 (PMC10979128; doi:10.12688/f1000research.132823.2)
Supplement: Peer review history

# Reviewer report: Reviewer response for version 2

**DOI:** 10.5256/f1000research.163228.r257448

**Published:** 2024-04-02

**Authors:** Simon Kaggwa

**Affiliations:** 1 Hult International Business School, Boston, Massachusetts, USA

Review at each Stage

**1. Title and Abstract**

 Title: The title accurately reflects the report's focus on cybersecurity in Zimbabwe's financial services sector. It is informative and concise.

**2. Introduction**

 The introduction effectively sets the context for the study, highlighting the importance of the internet in today's knowledge economy and the risks posed by cyberattacks. It provides relevant statistics and examples to underline the urgency of addressing cybersecurity in Zimbabwe. However, it would benefit from a clearer statement of the research questions or objectives towards the end to guide the reader on what the study specifically aims to address.

**3. Literature Review**

 The literature review is detailed and covers a wide range of topics relevant to cybersecurity, including the evolution of cyberspace, the impact of cybercrimes, and the state of cybersecurity frameworks worldwide and in Africa. While comprehensive, the review could be improved by organizing the themes more clearly and critically evaluating the sources. For instance, discussing the gaps in current research that this study aims to fill would strengthen this section.

**4. Methods**

 The methodological approach is well-detailed, explaining the survey design, participant selection, and data collection process. Ethical considerations are also addressed. However, the rationale for choosing a descriptive quantitative survey and the justification for the sample size and selection criteria could be elaborated on to enhance the method's section.

**5. Results**

 The results section presents findings on the ranking of cybersecurity constructs, the frameworks used by financial institutions, and barriers to effective cybercrime management. The inclusion of statistical data, such as means and standard deviations, is commendable. However, the presentation of these results could be improved with the use of tables or figures for a more straightforward interpretation of the data.

**6. Discussion**

 The discussion provides a good synthesis of the findings in relation to the literature review. It identifies key barriers to cybersecurity and suggests practical steps for improvement. However, the discussion could be strengthened by more directly linking the results to the specific objectives of the study and by offering a more critical analysis of the implications of the findings.

**7. Conclusion and Recommendations**

 The conclusion summarizes the main findings and their implications. Recommendations are practical and relevant. The call for future research is appropriate, although more specific suggestions for areas of investigation would be beneficial. The report could also benefit from a stronger emphasis on the limitations of the study, as acknowledging these can help contextualize the findings.

**Overall Assessment**

 This report provides valuable insights into the state of cybersecurity in Zimbabwe's financial services sector, highlighting significant challenges and offering practical recommendations. The authors have compiled a thorough analysis, though there are areas where clarity and depth could be improved, particularly in the abstract, methods justification, results presentation, and critical analysis in the discussion. Enhancing these areas would make the report an even more valuable contribution to the field of cyber security.

Is the work clearly and accurately presented and does it cite the current literature?

Yes

If applicable, is the statistical analysis and its interpretation appropriate?

Partly

Are all the source data underlying the results available to ensure full reproducibility?

Yes

Is the study design appropriate and is the work technically sound?

Yes

Are the conclusions drawn adequately supported by the results?

Yes

Are sufficient details of methods and analysis provided to allow replication by others?

Partly

Reviewer Expertise:

Finance and Cyber Securities case of America economies

I confirm that I have read this submission and believe that I have an appropriate level of expertise to confirm that it is of an acceptable scientific standard, however I have significant reservations, as outlined above.

# Reviewer report: Reviewer response for version 2

**DOI:** 10.5256/f1000research.163228.r257453

**Published:** 2024-03-28

**Authors:** Dinesh Kalla, Samaah Fnu

**Affiliations:** 1 Department of Computer Science,, Colorado Technical University, Colorado Springs, Colorado, USA; 2 Northeastern Illinois University (Ringgold ID: 2458), Chicago, Illinois, USA

This paper highlights the increasing sophistication of cyber threats, the limited skills available to combat these threats, and the challenges posed by emerging technologies. Maphosa emphasizes the need for financial institutions to establish a robust cybersecurity culture, invest in cybersecurity technologies, and employ specialized security professionals such as Chief Information Security Officers (CISOs) .Maphosa's study reveals that network and data security are the most highly ranked cybersecurity constructs, signifying their critical importance in safeguarding the sector. Conversely, physical security was ranked the least, suggesting a potential area for improvement or a shift in focus towards digital threats. Interestingly, the study found that a significant portion of financial institutions relied on the Information Technology Infrastructure Library (ITIL) and Control Objectives for Information and Related Technologies (COBIT) frameworks, with a noteworthy percentage yet to adopt any cybercrime frameworks .

 One of the paper's key recommendations includes the development of a national cybersecurity framework to enhance the strategic protection of Zimbabwean financial institutions. This framework should focus on creating a cybersecurity culture, addressing barriers to effective cybercrime management, and following best practices such as adopting established frameworks and appointing CISOs .The paper also underscores the importance of raising awareness about cybersecurity, suggesting that financial institutions collaborate with educational institutions to close the skills gap and enhance cybersecurity literacy. Furthermore, Maphosa calls for the government and private sector to partner in establishing Computer Emergency Response Teams (CERT) for the financial services sector, underscoring the critical need for improved cybersecurity infrastructure and readiness .

 In conclusion, "An Overview of Cybersecurity in Zimbabwe's Financial Services Sector" provides a comprehensive examination of the challenges and necessary steps to fortify the cybersecurity posture within Zimbabwe's financial services sector. The study's recommendations offer a roadmap for stakeholders to improve cybersecurity awareness, infrastructure, and policies, thereby enhancing the resilience of financial institutions against cyber threats .

Is the work clearly and accurately presented and does it cite the current literature?

Yes

If applicable, is the statistical analysis and its interpretation appropriate?

Yes

Are all the source data underlying the results available to ensure full reproducibility?

Yes

Is the study design appropriate and is the work technically sound?

Yes

Are the conclusions drawn adequately supported by the results?

Yes

Are sufficient details of methods and analysis provided to allow replication by others?

Yes

Reviewer Expertise:

Cybersecurity, AI , Big Data and Data Science

We confirm that we have read this submission and believe that we have an appropriate level of expertise to confirm that it is of an acceptable scientific standard.

# Reviewer report: Reviewer response for version 2

**DOI:** 10.5256/f1000research.163228.r255953

**Published:** 2024-03-25

**Authors:** William Vambe

**Affiliations:** 1 Walter Sisulu University, Mthatha, Eastern Cape, South Africa

The issues raised previously were addressed

Is the work clearly and accurately presented and does it cite the current literature?

Yes

If applicable, is the statistical analysis and its interpretation appropriate?

Partly

Are all the source data underlying the results available to ensure full reproducibility?

Yes

Is the study design appropriate and is the work technically sound?

Yes

Are the conclusions drawn adequately supported by the results?

Yes

Are sufficient details of methods and analysis provided to allow replication by others?

Yes

Reviewer Expertise:

IoT, Fog Computing, Cloud Computing, ICT4D

I confirm that I have read this submission and believe that I have an appropriate level of expertise to confirm that it is of an acceptable scientific standard.

# Reviewer report: Reviewer response for version 1

**DOI:** 10.5256/f1000research.145775.r211333

**Published:** 2023-10-20

**Authors:** Nomusa Dlodlo

**Affiliations:** 1 Rhodes University, Grahamstown, Eastern Cape, South Africa

Please refer to the attached file.

1. What is the total population of the people who you could pull the participants from and how large is the sample you interviewed. ow did you identify the sample- is it purposive sampling / targetted sampling?
2. What  frameworks  led to the questionnaire?
3. Be specific on the numbers. How many questionnaires were distributed. ? How many responses did you get back? Are the responses an adequate representative sample of the total population of potential interviewees. How did you choose who to distribute the questionnaire to? Was it intentional or random?
4. The discussion can be approached in sub-sections where you explain the results under each subsection and then give a comparison of the results with other similar researches, e.g., Adoption of cybercrime frameworks, sophistication of threats, technical skills, etc. You already have that. You just need to add the subtopics.

Is the work clearly and accurately presented and does it cite the current literature?

Yes

If applicable, is the statistical analysis and its interpretation appropriate?

Yes

Are all the source data underlying the results available to ensure full reproducibility?

No

Is the study design appropriate and is the work technically sound?

Partly

Are the conclusions drawn adequately supported by the results?

Yes

Are sufficient details of methods and analysis provided to allow replication by others?

Yes

Reviewer Expertise:

IoT, Blockchain, ICT4D, ICT in Education, ICT in Health

I confirm that I have read this submission and believe that I have an appropriate level of expertise to confirm that it is of an acceptable scientific standard, however I have significant reservations, as outlined above.

# Response

**Published:** 2024-02-10

**Authors:** Vusumuzi Maphosa

**Affiliations:** ICTS, National University of Science and Technology, Bulawayo, Bulawayo Province, Zimbabwe

Population: The study targeted IT experts from the country’s financial institutions comprising commercial banks, merchant banks, discount houses, building societies, and finance houses.

 Sampling: Convenience sampling was used to recruit participants.

 Framework: The top frameworks used are the Information Technology Infrastructure Library (ITIL) and Control Objectives for Information and Related Technologies (COBIT), while a fifth is yet to adopt cybercrime frameworks.

 Questionnaire: The study’s questionnaire was adapted from the International Organization for Standardization (ISO)/International Electrotechnical Commission (ISOC/IEC) ( ISOC/IEC, 2012). ISOC/IEC is a task force responsible for crafting and reviewing industry-wide cybersecurity standards every five years. 

 Questionnaire: An online questionnaire was used and 76 responses were received. The data set is available to the public. The distribution was random.

 Discussion: The discussion is categorised into cybersecurity, security of data, security issues in developing countries, and emerging threats.

 A paragraph on frameworks has been added to the discussion section.

 Developing countries have rapidly increased access to cyberspace, without corresponding effort to fortify cyberspace and improve security measures. Developing countries face challenges in adopting cybersecurity frameworks and struggle to deter cybercrime (Muller, 2015). The most common cybersecurity frameworks in Zimbabwe are the ITIL and COBIT, while other frameworks like the NIST and ISO 27002 are quickly gaining recognition.

 Muller, L. P. (2015). Cyber security capacity building in developing countries: challenges and opportunities Norwegian Institute of International Affairs, Norway.

# Reviewer report: Reviewer response for version 1

**DOI:** 10.5256/f1000research.145775.r211331

**Published:** 2023-10-09

**Authors:** William Vambe

**Affiliations:** 1 Walter Sisulu University, Mthatha, Eastern Cape, South Africa

The paper present a very important aspect especially with the incoming of 4IR, internet and where everyone now use mobile, online and internet banking daily. I have few issues that I think might improve the paper.

 Introduction

- In the context of Zim, is this statement true, "Decreasing data costs... ". Since the paper is about Zimbabwe, I think we need to contextualize why there is a rise in using online, mobile, internet banking.
- In your write up, I propose you present state of cyber security in the context of Europe, Africa, Sadc countries, then Zimbabwe in particular since it is the study area. This gives us a clear view. Then you conclude this section by giving us the aim./ objective of this paper. Last paragraph should be a signpost to inform the reader what to expect in the whole paper.

Literature

- In the literature/related work, I was expecting more focus and discussion to be on Zim not the world in general. Is there government policy, or financial organization policies pertaining to cybersecurity? What are the challenges and implications of implementing them? What does the current/literature say about cybersecurity in Zimbabwe? What are open gaps?
- These discussions will help us to see why there was a need to do this research and will also helps us to link with the results section.

Results

- I propose using graphs rather than tables as graphs show the comparison better.

**Since you are an expert, based on your results, I propose you put a section Possible Solutions/ Significant Recommendations, where you articulate what as a researcher think can be done to address the challenges you got from literature and your results. This can be backed by literature on solutions that have been implemented in other countries almost similar to Zim.

Is the work clearly and accurately presented and does it cite the current literature?

Yes

If applicable, is the statistical analysis and its interpretation appropriate?

Partly

Are all the source data underlying the results available to ensure full reproducibility?

Yes

Is the study design appropriate and is the work technically sound?

Yes

Are the conclusions drawn adequately supported by the results?

Yes

Are sufficient details of methods and analysis provided to allow replication by others?

Yes

Reviewer Expertise:

IoT, Fog Computing, Cloud Computing, ICT4D

I confirm that I have read this submission and believe that I have an appropriate level of expertise to confirm that it is of an acceptable scientific standard, however I have significant reservations, as outlined above.

# Response

**Published:** 2024-02-10

**Authors:** Vusumuzi Maphosa

**Affiliations:** ICTS, National University of Science and Technology, Bulawayo, Bulawayo Province, Zimbabwe

Developed countries have developed effective cybersecurity frameworks and policies to strengthen operations, raise awareness and support training programmes (Russell et al., 2017). Countries such as the United States of America, Australia, Canada and South Africa use cybersecurity to secure and fortify critical infrastructure which drives socioeconomic development (Catota et al., 2019). Developing countries lag in the adoption of cybersecurity despite the unprecedented adoption of ICTs over the last two decades. Only 11 African countries have cybersecurity policies (Kshetri, 2019). Kabanda (2019) noted that cybersecurity systems in Africa are underdeveloped due to limited infrastructure, lack of funding, inadequate policies and legislation, lack of education and awareness, and limited reporting and data-sharing platforms.

 In Zimbabwe, not much has been done in terms of cultivating a culture of cyber security and combating cybercrimes. Zimbabwe has a massive shortage of cybersecurity specialists and this is compounded by the lack of frameworks and policies to drive national implementation programmes. Kabanda (2019) notes in Zimbabwe, cybersecurity is regarded as an afterthought and is not part of the core business strategies and this is worsened by the unprecedented brain drain of skilled cybersecurity personnel. Zimbabwe faces challenges such as a lack of programmes and opportunities to equip the general public with skills, knowledge and awareness to fight cybercrime (Mutunhu et al., 2022). The Cyber Security and Data Protection Bill promulgation is a critical step in fighting cybercrime, but it has been widely criticised and viewed as a tool for the State to obstruct civil society, and the media in the fight against corruption (Transparency International, 2020). There is a need to include non-state actors in the development of the Bill.

 Zimbabwe mobile data prices have been decreasing over the past two decades from USD180.00 per giga byte in 2010 to USD15.50 in 2020 (POTRAZ, 2021).

**Recommendations**

 More awareness and education are required for cyberspace users. A cybersecurity culture should be developed in the early stages of schooling such as at the primary school level.

 There should be a deliberate effort to grow cybersecurity skills which are extremely important to the financial services sector and are in short supply due to brain drain.

 The government and the private sector should partner to set up Computer Emergency Response Teams for the financial services sector and other sectors.

 The cybersecurity bill should be flexible to allow for continuous review by state and non-state actors and align with the dynamic threat levels.

 Tax rebates on cybersecurity equipment are required to ensure that organisations can afford basic cybersecurity equipment.

**Works Cited**

 Catota, F.E; Morgan, M.G; Sicker, D.C. (2019). Cybersecurity education in a developing nation: the Ecuadorian environment. 1–19. DOI:10.1093/cybsec/tyz001

 Kabanda, G. (2019). A Bayesian Network Model for a Zimbabwean Cybersecurity System. *Orient. J. Comp. Sci. & Technol., 12*(4), 147-167.

 Mutunhu, B; Dube, S; Ncube, N; Sibanda, S. (2022). ****Cyber Security Awareness and Education Framework for Zimbabwe Universities. A Case of National University of Science and Technology**** Proceedings of the International Conference on Industrial Engineering and Operations Management, Nsukka, Nigeria

  Russell, J.D; Weems, C.F; Ahmed, I; Richard III, G. (2017). Self-reported secure and insecure cyber behaviour: factor structure and associations with personality factors. J Cyber Secur Technol. 1 (3-4), 163-174.

 POTRAZ. (2021). Mobile data prices in Zimbabwe. cyber behaviour: https://t3n9sm.c2.acecdn.net/wp-content/uploads/2021/02/POTRAZ-Press-Statement-Mobile-Data-Prices-in-Zim-Final.pdf, Harare, Zimbabwe.

 Transparency International. (2020). Zimbabwe: Cyber Security and Data Protection Bill would restrict anti-corruption watchdogs. https://www.transparency.org/en/press/zimbabwe-cyber-security-and-data-protection-bill-would-restrict-anti-corruption-watchdogs-1
